# Hospital Meetings

**Published:** 1901-04-06

**Authors:** 


### The Institutional Workshop.

### HOSPITAL MEETINGS.

### ROYAL HOSPITAL FOR DISEASES OF THE CHEST.

The Lord Mayor (Mr. Alderman Green) presided at the eighty-seventh annual Court of Governors of the Royal Hospital for Diseases of the Chest, City Road, on Wednesday, March 20th. He moved the adoption of the report of the Council and the balance-sheet for 1900, from which it appeared that the ordinary income was £6,696 and the ordinary expenditure £7,702. The extraordinary income (legacies) was £669. The outlay thus exceeded the total revenue by £336. As compared with the previous year there was a falling-off in income of £997, chiefly due to a decrease of £964 in the receipts from legacies. Annual subscriptions showed a decrease of £37, but donations (which included one sum of £1,000 from Miss Thompson, at whose wish a bed had been dedicated to the memory of the late Dr. Hodgkin) showed an increase of £321, and totalled £3,440. The expenditure showed an increase on 1899 of £211, due to the increased cost of gas and coal and provisions generally; reductions having been effected under the headings of management, finance, salaries, wages, &c. The in-patients numbered 702 as against 798 in 1899, and the new out-patients 6,558 as compared with 6,671. At a special meeting of the Council held in January resolutions of condolence with the King were passed on the death of her late Majesty, who for upwards of 50 years was the patron of the Hospital, and petitioning the King to become in turn their patron. The Prince of Wales's Fund and the medical staff of the hospital having pressed on the Council the necessity of providing improved sleeping accommodation for the nursing staff, it had been decided to have a festival dinner on the 6th May next. Sir Andros de la Rue had consented to preside, and their object would be to raise £10,000 to purchase a freehold site adjoining the hospital for which they were already in negotiation, and thereon to build a nurses' home. The death was reported of Mr. Charles J. Webb, for many years chairman of the house committee.

In recommending the adoption of the report, the chairman reminded the meeting that that hospital was the first established in Europe for the study of consumption and other diseases of the chest, and he was quite certain that great interest was taken in the movement throughout Europe with regard to the treatment of tuberculosis. He learned that in July a very large meeting was to take place on the subject and he hoped benefit would arise from that conference. He was glad to notice that some of the City Companies contributed to the funds, and ventured to hope that more of them would do so. He would like to see a dozen or more subscribing regularly.

The motion was adopted. The Hon. Claude Hay, M.P., moved that the thanks of the meeting be given to the president and officers for their services during the year, which was seconded by Dr. Arthur Davies and carried. Thanks were also voted to the Council of Management, and Dr. Philip Hensley, Mr. T. A. F. Kingscote, Sir Patteson Nickalls and Mr. Willoughby Smith were elected to that body, the election of Mr. Hay and Mr. W. P. Fuller being confirmed. Thanks having been given to the hon. medical officers and to the chairman the Court adjourned.

### LONDON TEMPERANCE HOSPITAL.

The twenty-eighth annual meeting of the London Temperance Hospital was held on Thursday, March 21st, Alderman Vezey-Strong presiding. After passing a special resolution deploring the national loss occasioned by the death of her late Majesty, the reports of the Board of Management and of the Treasurer for 1900 were read and adopted. From these it appeared that the ordinary income was £6,297 and the ordinary expenditure £10,585. The extraordinary income was:—Legacies, £7,323, and the late Mr. George Sturge's Trustees (final payment) £1,000, together £8,323. The extraordinary expenditure was £347 for repairs and sanitary and engineering work. The in-patients during the year numbered 1,282, 72 less than in 1899. The deaths numbered 117, of which 32 occurred within 24 hours of admission. The out-patients numbered 8,327, with 21,015 visits. Casualty cases numbered 14,012, with 32,361 visits. In November last Dr. Nicoll, having completed his term of service as resident medical officer, retired and was replaced by Dr. Rhodes. There were three cases recorded during the year under the rule which provides that alcohol may be administered in exceptional cases. Of these two died. The committee affirmed that after the experience of upwards of 27 years, the results obtained by the treatment of disease without the ordinary use of alcohol were at least equal to those shown by other general hospitals. The support of the medical profession was therefore claimed to a practice which, while not hindering recovery, protected patients from the risk of evil attending even the medicinal use of alcoholic liquors, and tended to explode the superstitious value so long attached to alcohol as a therapeutic agent; and which if generally adopted would relieve the funds of hospitals from a useless expenditure which might be applied to augment real comforts. The report was adopted, and Mr. Thomas Cash and Mr. A. F. Hills were re-elected president and treasurer respectively, other routine business being transacted.

Public Meeting.

His Grace the Archbishop of Canterbury presided at the subsequent public meeting, which was largely attended. He excused himself for being a few minutes late on the plea that he had waited for a division in the House of Lords on the Bona-fide Travellers Bill. They had not won, he said, but they had got within five of winning, and that would surely have results, for the Government did not like being in a majority of five only in the House of Lords, especially after issuing a strong Whip against the Bill. However, he thought that hospital was as important in its bearings on the temperance question as any Bill they could pass, for they were able there to teach not only the general public, but medical men, that alcohol was not so necessary in the treatment of disease as had been thought. It was believed for a great number of years that alcohol was not only a very efficacious drug, but an indispensable one. They had quite disproved that. They did not say there that they would not give alcohol under any circumstances, but only when satisfied that it was necessary to try it. And that was a rule which diminished the quantity used in a very marked degree indeed. They had come to the conclusion, from careful observation, that the use of alcohol was as a rule mischievous, and they reserved its use for such cases as the medical men decided it must be employed in. It was clearly possible for the medical profession to separate those extreme cases where it must be employed from the doubtful cases, and those in which it had probably better not be given at all. At one time the medical profession did not believe in temperance at all, and some of the reputable medical papers even spoke of trying a physician at the Old Bailey if he allowed a patient to die without having given alcohol. But now the medical profession were pretty well agreed that alcohol, as a general rule, was no good at all to the human body, and there seemed no doubt that one was more likely to keep healthy by abstaining entirely than by any other means one could adopt. They did not claim that total abstinence was absolutely necessary for good health, but they could bring forward many good instances of it. He was inclined to regard himself as a good example. He was a total abstainer and he was close on 80, and he doubted if many men could be found who could do so much as he was obliged to do in his position. He doubted if there were many harder working men in the country. And there were many others who could bear similar testimony. It was sometimes said there was risk in breaking off the use of alcohol after one had grown accustomed to it; but there again he was an example to the contrary since, though he never was drunk in his life, yet it was not till he was 50 that he gave up the use of it, and after 30 years' experience he could say without hesitation it was the wisest thing he ever did in his life. Of course, if his physician ordered it he should take it, just as he would take calomel or any other drug—unless he mistrusted his physician, in which case he should change him. But nowadays if a man told his doctor he was a total abstainer the doctor accommodated himself to that, and prescribed something else which did equally well or better. They no longer said they could not bring one back to health without it. They had gradually learned the lesson that hospital had to teach. It had been a difficult lesson in the first place because the men of science had had to learn from the men of no science. But the medical profession were always really open to scientific argument. The reason they had held out so long was that they had a long-standing tradition, for centuries past, that this was a very useful drug both in health and disease. And medical science had so many branches that the study of the whole human body was a very large study indeed, and they must not wonder if progress seemed slow. As a matter of fact it had of late years been very rapid. Many things were done now which would have seemed miracles not long ago. They knew much more of the causes of disease than they did a while back. Looking over the past 100 years, both in surgery and in medicine, the progress was quite astounding compared with what it had been up to that date. All honour then to those who in that hospital had set aside traditions that must go back 1,000 years at least. What they achieved in that hospital enlightened medical men in the way that suited the case best, because even the stupidest doctor liked what science he had to be accurate.

Other speakers, among whom were Mr. Wm. Johnston, M.P., and Mr. J. Herbert Roberts, M.P., followed.

### ROYAL LONDON OPHTHALMIC HOSPITAL

Lord Avebury, the President, took the chair at the annual general meeting of the Governors of the Royal London Ophthalmic Hospital on Wednesday, March 20th. He moved the adoption of the report of the Committee of Management and accounts for the past year. From these it appeared that five wards, containing 68 beds, still remained unopened for want of funds, so that out of 138 beds there had only been 70 available, and many patients who should have been taken in could not be admitted. The daily average of beds occupied was 64, the in-patients numbering 1,871. The out-patients numbered 36,932, with 99,821 attendances. The ordinary income was £5,088, and the ordinary expenditure £10,785. The extraordinary income was put down as £3,585, made up of donations through appeal made through newspapers £490, special appeal £1,845, and Prince of Wales's Fund £1,250. There were also legacies amounting to £1,097. The deficiency on the year, after crediting the legacies, was £1,014. This, added to the deficit at the end of 1899, made a total deficiency on 31st December last of £2,592. The most notable item of expenditure was the enormous increase of rates, which, for the half-year begun in October, were on the basis of £960 per annum. For nearly a century the valuable property of the hospital in Moorfields was assessed at a nominal amount only by the City of London Union, but on the occupation of the new building, which was in the Holborn Union, a different course was taken, and the rates now amounted to nearly one-eleventh of the annual expenditure. In September the hospital was £5,000 in debt; the rates could not be met; a summons was served, and payment was ordered within 14 days. The case was widely reported and commented on in the Press, and the money speedily subscribed. During the year Messrs. Alan Cadell, Stanley Christopherson, William Muir, Edward Pollock, C. A. Galton, and V. I. Chamberlain were elected on the Committee of Management. The death of Mr. Edward Hogg was recorded with much regret, as was also the resignation of Mr. F. H. Janson. Mr. Edward Pollock had also resigned in consequence of official duties. An old and staunch supporter had also been lost by the death of the chaplain, Prebendary Whittington. The vacancy was filled by the appointment of the Rev. Dr. Walters. Owing to the great increase in the number of out-patients a joint committee of members of the Management Committee and the Medical Board appointed to consider the matter recommended three additional appointments, and as a consequence Mr. Percy Flemming, Mr. J. H. Fisher, and Mr. Arnold Lawson were elected. Mr. E. Treacher Collins and Mr. W. T. Holmes Spicer had also been appointed surgeons. Great regret was expressed at the resignation of Mr. John Tweedy, who had been surgeon to the hospital for 21 years. He had, however, accepted the post of consulting surgeon. The consequent vacancy on the surgical staff had been filled by the appointment of Mr. C. Devereux Marshall as assistant surgeon. The inquiry officer appointed in 1893 had been obliged during the year to refuse 448 applicants, as being considered able to pay a surgeon. Being most reluctant to curtail the work of a hospital which had been going on for over 100 years, which had a world-wide reputation, and which relieved 40,000 patients annually, the committee made a special appeal for help. The new building was perfectly designed and equipped for its special object, and worthy of the work and teaching of the oldest and largest eye hospital in Great Britain.

The Chairman briefly touched upon the chief points in the report, which was seconded by Mr. Sturgess and carried.

Messrs. A. Gordon Pollock, Herbert Samuelson, F. C. Scotter, and H. P. Sturgis were re-elected to the committee; the election of Mr. C. A. Galton and Mr. V. I. Chamberlain was confirmed; and a motion was passed recording the deep sense of the loss sustained by the death of Mr. John Deacon, who had held the position of treasurer for the past nineteen years.

Votes of thanks to the medical, nursing, and secretarial staffs, to the newspapers for their support, and to the Chairman for presiding were adopted, and the meeting separated

### THE NORTH LONDON HOSPITAL FOR CONSUMPTION.

The annual Court of Governors of the North London Hospital for Consumption was held on Wednesday, March 20th. at 41 Fitzroy Square. Mr. F. D. Mocatta was in the chair. The report stated that 493 patients had been received during the past year; these, with 80 remaining on December 31st 1899, and 384 renewals, made a total of 957. Since the open-air extension was opened in June, 1900, patients had been sleeping on the balconies in the open; and even through November and December they had benefited by this treatment. In this matter the North London Hospital was in the vanguard of advance, for even the Continental Sanatoria knew but little of this. The balconies, which had a south-west aspect, accommodated 12 male and 12 female patients.

Phthisical cases complicated with bronchitis or recent pleurisy were not deemed suitable, nor, during the cold weather, were old or rheumatic subjects, and all patients were on probation for awhile in the open-air wards. Blankets were provided almost *ad libitum*, as well as hot-water bottles but no attempt was made to artificially heat the balconies. The marked immediate effect on the phthisical patients, whether in the balconies or in the open-air wards, was very noteworthy; appetite and weight increased, sleep improved, and fever, in the great majority of cases, was soon absent. Could those patients who were discharged at the end of nine or twelve weeks, and who were classified as "much improved" or "improved" remain under treatment for months instead of weeks, there was no doubt that many cases might be discharged as cured. Of 332 cases of phthisis under open-air treatment, 103 were much improved, 130 were distinctly improved, 35 were slightly improved, 40 left the hospital in practically the same condition in which they were when admitted, 21 became worse, and 3 died. It was clearly shown that the patients in whom the disease was least advanced derived the greatest benefit; in practically one-third of those subjected to the treatment the disease was decidedly advanced, while only about a quarter could be described as early cases. There had been 3,839 out-patients at Hampstead and Fitzroy Square.

In moving the adoption of the report and balance-sheet, Mr. Mocatta said that the hospital not only maintained its high standard of efficiency, but it had advanced to a higher point, it was both stronger and larger that ever before. There were now 100 beds, while there were 200 accepted applicants on the books. The experiment was succeeding beyond their best hopes, and it was an encouraging fact that other countries were studying and adopting the treatment. A Nurses' Home was much needed, and it was hoped at some future time to provide one in the grounds; the present arrangement of renting a house outside was a temporary one. Although not in a condition of distress, the hospital required the sum of £5,000; and for his own part, he would rather have a moderate income from living people than a large one from legacies; intelligent sympathy and work were at least as valuable as money. There was nothing more to be desired than a Convalescent Home, where the treatment received in the hospital could be continued; from the infectious nature of the disease, phthsical patients were debarred from the ordinary Convalescent Home: they had already been the means of restoring many to health and—of all things to be desired—work; and a Convalescent Home would be of the very greatest service in completing the cure inaugurated at the hospital.

Mr. Stedall said that the wide scope of the hospital was proved by the fact that patients had been received from Scotland, Canada, the West of England, Lancashire, &c., while five beds were always occupied by patients from Yorkshire.

### ST. PETER'S HOSPITAL.

Mr. F. A. Bevan, the treasurer, presided at the thirty-third annual meeting of governors and subscribers of St. Peter's Hospital for Stone on March 26th. The report of the Committee of Management stated that during the past year 548 in-patients were admitted, being 14 less than in 1899. The number of beds occupied was, however, larger, owing to the greater severity of the cases necessitating a longer stay. Of these 497 were discharged cured or relieved, 31 unrelieved, and 20 died, some of the last having been sent in in a very enfeebled condition; 87·4 of the patients admitted were subjected to operation; 15 deaths followed operation; and the death rate of operated cases was $3\frac{1}{8}$ per cent. The number of out-patients seen was 4,387, and the attendances registered 40,725. The voluntary contributions of out-patients totalled £2,082—considerably the largest item in the income of the institution. The extensive structural alterations to the out-patient department carried out last year had materially increased the facility and efficiency of the work and added to the comfort of the patients; but though rendered necessary by the progress of aseptic surgery, these changes had been carried out at an expense which had seriously crippled the resources of the hospital. During the year there had been a considerable increase in the attendance of medical men on the practice, the number being 823 as against 728 in 1899. Upon the retirement of Mr. Irwin Hunter, who had held the post of house-surgeon for the past twelve months, Mr. Herbert Franklin, M.R.C.S., was elected. The committee reported with great regret the death of Mr. Francis Rayworth Heycock, who had been connected with the hospital as honorary and consulting surgeon since 1873. The ordinary income for the year was £3,630, and the ordinary expenditure £3,889. The extraordinary expenditure on building improvements, &c., amounted to £203. The excess of expenditure over receipts was thus £462.

In moving the adoption of the report, the Chairman said it would be seen that almost all the figures correspond with those of the previous year, and the hospital had been maintained in the same state of efficiency. But they had again overspent themselves, as in the previous year, by between £400 and £500, and if they went on much longer at that rate they would soon have paid away all their reserve fund and be very hard up indeed. But they did not see how they could act differently; their patients were as numerous as ever, and patients who could be relieved often had to wait because they had no room for them, with great risk to their chances of ultimate cure. The pressure on their accommodation, both in the out-patient and in-patient departments, showed how the hospital was appreciated; and when they looked at the results achieved they saw the reason why. The cases were treated with such remarkable skill that nearly everyone went away cured or relieved. Their death-rate was only 3·03 per cent., and considering the nature of the cases this was infinitesimal. This was a great contrast with what used to be the case. When they looked back to the start they saw that the number of deaths was 18 out of 118 cases; now it was 15 out of over 500; and that showed what a benefit the hospital had been to the community. They had been instrumental in giving such education to the gentlemen who undertook the work that they were now able to undertake operations with almost a certainty of success. They claimed to benefit people to an extent not attained at other places. For one thing the number of men who came here and got relief in the evening, being thus able to attend to their work in the daytime, was a great benefit to the community. He thought, therefore, they might appeal to the City companies to help them out of debt. The war was unhappily still dragging on, and now there was the Memorial Fund being raised at the Mansion House. But in spite of these new claims, old-established charities should not be neglected. The results they were obtaining were never more satisfactory; the hospital was never more appreciated; the skill and attention of their surgeons were never greater. They could do more if they had more room, but for the present they must be content to keep on in their present scale. When the war was over, and the income-tax going down instead of up, they must try to extend their work.

The motion was seconded by Mr. Galsworthy, who suggested that they should sell Consols to the extent of half their debt of £800. He thought they could then appeal with greater force for help to wipe out the rest.

The report was unanimously adopted, as was that of the surgical staff, moved by Mr. Hurry Fenwick, who, in returning thanks for a complimentary vote to the staff, mentioned that, not only was the number of surgical cases passed through their beds twice that of other hospitals, but they had numerous cases sent them from general and from special hospitals for special diagnosis, these being then sent back for the operation advised. This showed the way that the hospital was looked at, not only by the medical profession, but by other hospitals as well.

The Committee of Management was re-elected, with the addition of the names of Commander Crutchley and Mr. S. Waring, and a vote of thanks to the Chairman brought the proceedings to a close.

### WEST LONDON HOSPITAL.

Mr. Val. Prinsep, R.A., presided at the forty-fourth annual meeting of the West London Hospital, Hammersmith, on March 25th. In moving the adoption of the report he said he thought every Englishman must be proud of institutions of that kind. When travelling abroad they saw in many cities and countries sumptuous institutions, nobly housed and magnificently provided for; but unfortunately these were almost entirely maintained by Government. He was glad that in this country hospitals were maintained by private enterprise, because our greatness as a nation had mainly been built up by the individual effort of our merchants and others, who showed that our commerce could get on without aid from the Government. They might say there was hardly a hospital in England that was not greatly in debt. It was unfortunate perhaps in some ways that it should be so, but he thought they might look at it also from the point of view that in raising these buildings they were discounting the gratitude of the future. As a nation they could not get on without a national debt, and in the same way a little debt was a good thing for a hospital. But it should not be too much, and as a fact in that hospital they wanted more money to extend their work. They wanted £13,000 for a nurses' home, a laundry, and other matters, and about £14,000 for an administration block which should complete the frontage on to the Hammersmith Road. He had been told by a member of the Prince of Wales's Hospital Fund Council that they had not given that hospital as much as he could have wished because there were certain organisations not complete. That seemed to him a very stupid policy, because they were not complete simply because they had not money to complete with. They had applied to His Majesty to be pleased to continue his patronage, and he was glad to be able to read a letter from Sir Dighton Probyn acceding to that request.

From the report and accounts presented it appeared that the ordinary income was £6,843 and the ordinary expenditure £8,912. The extraordinary income — legacies and awards from charity bequests — was £2,962 and the extraordinary expenditure £47. Regret was expressed at the death of the late Bishop of London, who was last year elected a Vice-President, and of Colonel Stracey Clitherow, a member of the Board. The Rev. Anastasio Forrest and Mr. George W. Dennis had been elected to the Board. The death of the Consulting Physician Accoucheur (Sir William Priestley) was also reported, and the resignation, after nearly seven years' tenure of office, of Assistant Physician Dr. Wm. Aldren Turner. The staff of assistant-physicians had been completed by the election of Dr. Henry John Davis. Messrs. Ernest Wool Lewis and George P. Shuter had been re-appointed assistant-administrators of anæsthetics for a further period of one year. In consequence of the absence of Assistant-Surgeon Mr. G. L. Cheatle at the war in South Africa, Mr. Aslett Baldwin was appointed acting assistant-surgeon for twelve months certain, from February 12th, 1900. The resignation of the housekeeper, Miss Ann I. Goodchild, after nearly 10½ years' service, had led to the abolition of that office and the creation of the laundry as a separate department under a superintendent, subject to the Matron. Miss Florence Ethel Nevile had been appointed Assistant-Matron. In the person of Sister Mary Thomas the nursing department had lost one whose services to the hospital had covered a period of nearly 27 years, and the Board considered her connection with the hospital was of so exceptional a character as to warrant the presentation of a sealed testimonial illuminated on vellum and supplemented by a purse of 16 guineas. The Medical Council also presented her with a testimonial, together with a purse containing £43. It was regretted that the financial position of the hospital was not held to justify the grant of a pension. An efficient X-ray apparatus had recently been installed and placed in charge of Assistant Physician Dr. H. J. Davis. The Pathological Laboratory had been active and useful: 557 specimens had been examined, of which 169 were submitted by outside practitioners. The advantages offered to students by the West London Postgraduate College, attached to the hospital, had continued to attract members of the medical profession, not only from the ordinary area served by the hospital, from which no fewer than 27 became life members during the year, but also from the Royal Navy and the Indian Medical Services and from the colonies; 229 members had been enrolled, being an increase of 66 upon the number last reported; while the courses of study taken out numbered 371, or an increase of 106 upon last year's figures. At the opening of the autumn session the first conversazione was held by the college, at which the attendance of members was numerous, and the proceedings passed off in a manner most gratifying to all concerned. The West London Medico-chirurgical Society had continued to maintain its headquarters at the hospital; its membership was maintained at 570. The in-patients numbered 1,973 as compared with 2,042 in 1899. The average number of beds occupied was 123·81, as against 124·16. The average weekly cost per patient was £1 2s. 5¼d., as against £1 3s. 4½d., and the average total cost per patient £3 13s. 5½d., as against £3 14s. 1½d. The cost per occupied bed was £58 10s. 6½d., as against £60 19s. 3d. in 1899, and this improvement followed one of a rather larger amount on 1898, the saving in the two years amounting to £5 3s. 8½d.

The report was adopted; and various resolutions of thanks having been cordially recommended and carried, the proceedings terminated.

### ROYAL SEA BATHING HOSPITAL.

Mr. Michael Biddulph, M.P., the treasurer, presided at the 110th annual Court of Directors of the Royal Sea Bathing Hospital on the 25th March ultimo. In moving the adoption of the report he said the institution was in an admirable state of efficiency, but they were short of funds. A number of repairs and improvements had been carried out: these were very beneficial, but they had cost money, and the year closed with a deficit of close upon £3,000. If some kind friends did not come forward and replenish their empty exchequer he was afraid they would either have to break in on their capital by selling out some of their investments or reduce the number of patients admitted. But, of course they were very reluctant to do either; and considering the claims the institution had on the charitable, and the very high recommendations for their efficiency and the results obtained, he hoped they would be able to avoid such a step. They had approached His Majesty the King to consent to be the patron of the hospital in the place of her late Majesty, who had filled the office in succession to George III., George IV., and the Duchess of Kent; he was glad to say that that consent had been accorded.

The report presented gave the number of in-patients during the year as 632. There were 31 out-patients. Regret was expressed at the death of the Rev. Prebendary Whittington and of Major Bell, for many years a member of the Executive Committee. Sir Samuel Wilks had retired from the Medical Board, to which body had been added Dr. W. Cayley, of the Middlesex; Dr. T. H. Green, of the Charing Cross; and Mr. Edmund Owen, of St. Mary's Hospitals. Mr. Peirce, who for eighteen years held the office of secretary, left last October to take up the office of secretary-superintendent to the Hereford Infirmary. His place had been filled by Mr. A. Nash from St. Thomas's. The ordinary income for the year was £5,174, and the ordinary expenditure £8,129. The extraordinary income (legacies) amounted to £1,066, and the extraordinary expenditure (repairs, building improvements, &c.) to £1,098. The report was adopted; and some routine business having been done, the meeting separated.

